# Milk-Based Nutraceutical for Treating Autoimmune Arthritis via the Stimulation of IL-10- and TGF-β-producing CD39^+^ Regulatory T Cells

**DOI:** 10.1371/journal.pone.0117825

**Published:** 2015-01-28

**Authors:** Massimo Maddaloni, Irina Kochetkova, SangMu Jun, Gayle Callis, Theresa Thornburg, David W. Pascual

**Affiliations:** 1 Department of Infectious Diseases & Pathology, University of Florida, Gainesville, Florida, 32611, United States of America; 2 Department of Microbiology & Immunology, Montana State University, Bozeman, Montana, 59717, United States of America; Northwestern University Feinberg School of Medicine, UNITED STATES

## Abstract

Autoimmune diseases arise from the loss of tolerance to self, and because the etiologies of such diseases are largely unknown, symptomatic treatments rely on anti-inflammatory and analgesic agents. Tolerogenic treatments that can reverse disease are preferred, but again, often thwarted by not knowing the responsible auto-antigens (auto-Ags). Hence, a viable alternative to stimulating regulatory T cells (Tregs) is to induce bystander tolerance. Colonization factor antigen I (CFA/I) has been shown to evoke bystander immunity and to hasten Ag-specific Treg development independent of auto-Ag. To translate in treating human autoimmune diseases, the food-based *Lactococcus* was engineered to express CFA/I fimbriae, and *Lactococcus*-CFA/I fermented milk fed to arthritic mice proved highly efficacious. Protection occurred via CD39^+^ Tregs producing TGF-β and IL-10 to potently suppress TNF-α production and neutrophil influx into the joints. Thus, these data demonstrate the feasibility of oral nutraceuticals for treating arthritis, and potency of protection against arthritis was improved relative to that obtained with *Salmonella*-CFA/I.

## Introduction

Rheumatoid arthritis (RA) is a chronic, systemic inflammatory disorder affecting as much as 0.24% world-wide and as much as 1% of the US population [1,2]. The exact frequencies depend on geographical origin and gender, women being three times more affected than men [2–4]. While many organs are affected, the main targets of RA are synovial joints, and approximately half of the affected patients become disabled over the progression [5]. RA is mediated by T cells, predominantly CD4^+^ T cells. Collagen-induced arthritis (CIA) can be induced upon immunization with heterologous collagen II (CII) in DBA/1 mice, and it serves as a model for RA [6]. CIA and RA share many critical features of the disease pathogenesis, including CD4^+^ T cells mediated inflammation with extensive cartilage and bone damage, resulting in joint deformities [7,8]. This similarity is commonly exploited to use CIA as a model for RA and as a tool to investigate novel approaches to prevent and treat RA. Currently, treatments vary in nature. Non-pharmacological approaches, e.g., physical and nutritional therapies, are palliative and do not stop the progression of the disease, whereas pharmacological therapies relying on analgesia and anti-inflammatory drugs, including steroids, have deleterious side-effect particularly in the long-term care [9,10].

An interesting alternative for treating autoimmune disease that is receiving attention relies on the complex interactions between the host and its microbiota. It is believed that the association between the vertebrate hosts and the microbiota arose as an association that would simultaneously enhance host digestive efficiency and ensure a steady nutrient supply for the microbes [11]. The immune system has evolved innate mechanisms to prevent microbes from invading the exposed epithelia of the mucosa and the skin. Understanding how to manipulate the host innate immune system with probiotics provides an alternative strategy to inflammatory diseases rather than reliance on traditional pharmacological interventions.

Associated with various mucosal compartments, lactic acid bacteria (LABs), are important beneficial contributors to the host microflora and aid in maintaining immune homeostasis [11]. They have the capacity to either trigger or to silence the immune system, which appears to be strongly species- and strain-specific. How this occurs is dependent on the host’s pattern recognition receptor the LAB stimulates, which in turn results in varied immune responses. While LABs are traditionally used for their probiotic properties [12], genetically engineered LABs have been designed to produce and deliver therapeutic heterologous proteins to the host mucosa. One commonly used application for LABs is their potential for vaccine delivery to protect against infectious diseases and toxins [13]. Its second application is the delivery of immunotherapeutics to treat inflammatory diseases [14]. It is this latter property which is the emphasis of this report.

Colonization Factor Antigen I (CFA/I) fimbriae from the Gram-negative *Escherichia coli* are encoded by an operon containing four structural genes whose products are all secreted for assembly of the fimbriae on the outer membrane [15]. CFA/I fimbriae are heteropolymers composed of a minor adhesive subunit (tip protein), CfaE, joined ~1000 major subunits of CfaB. Assembly is directed by CfaA, a periplasmic chaperone which promotes proper folding, polymerization, and delivery to an outer membrane usher protein, CfaC, which extrudes the subunits and also acts as a platform to ensure proper assembly of the helix. Here we describe the reengineered *cfaI* operon for suitable expression by Gram-positive *Lactococcus lactis* subsp *lactis* IL1403 under the control of a tailored composite, semi-synthetic promoter. The recombinant lactococci grown on either standard culture medium or in milk, were orally fed to mice with CIA. We show that in both instances, *L*. *lactis*-CFA/I remarkably ameliorates arthritis with cytokine profiles consistent with an induced anti-inflammatory response supporting the observed clinical intervention.

## Materials and Methods

### Ethics Statement

All animal care and procedures were in accordance with institutional policies for animal health and well-being and approved by Montana State University Institutional Animal Care and Use Committee (IACUC) under protocol 13 and approved by University of Florida IACUC under protocol 566.

### Bacterial Strains and Media

*Lactococcus lactis* subsp. *lactis* IL1403 (IL1403) was grown on M17 plus 0.5% glucose (M17G) or in commercially available ultra-pasteurized whole milk. Although various brands of milk supported growth, only in Organic Valley milk did IL1403 routinely achieved densities of 2–4×10^7^ CFUs/ml versus 5–10×10^8^ CFUs/ml in M17G. To increase the bacterial density similar to that in M17, milk was fortified by adding 1% peptone, 1% glucose, and 2g/L β-glycerophosphate pH 7.

### Molecular Engineering of *L*. *lactis*

Plasmids were maintained in *E*. *coli* TOP10 and manipulated according to standard procedures. Restriction enzymes, T4 ligase, Taq polymerase (Phusion) were purchased from New England Biolabs (Ipswich, MA USA). The construction of pBzMM153 is synthetically described hereafter with the *cfaI* operon [16] under a tripartite, semi-synthetic promoter designed *in silico* for this investigation and borne on the pMSP3535H3 [17] or pIB184 [18] backbone. Plasmids were maintained in *E*. *coli* TOP10 and manipulated according to standard procedures. Restriction enzymes, T4 ligase, Taq polymerase (Phusion) were purchased from New England Biolabs. Synthetic sequences were hand-designed, hand-codon optimized when necessary, and they were purchased at Genescript. Sequencing was performed by University of Montana Murdock DNA Sequencing Facility (Missoula, MT). The construction of pBzMM153 is synthetically detailed hereafter. Each of the four genes was fused to a different lactococcal secretion signal peptide (SP) to avoid recombination, namely: usp45 (GenBank: ABY84357.1), PrtP (NCBI Reference Sequence: NP_858119.1), Exp4 (GenBank: AAC14604.1), lac (GenBank: AAA64243.1). A synthetic mini-operon, encoding for *cfaB* and *cfaE* framed to usp45 and PrtP signal peptides (SP), respectively, was designed to have AgeI and SmaI sites at the outer ends. This construct was named pBzMM122, and it featured an optimal Shine-Dalgarno sequence at the 5′ end, right upstream usp45-cfaB, and an 8-cutter PmeI restriction site in between usp45-cfaB and PrtP-cfaE. A separate synthetic gene was designed to have Exp4 SP framed to *cfaA* followed by the lactamase SP, at the 3′ end of which there is a NheI restriction site. *cfaC* was amplified from *Salmonella* H696 [16] with primers that deleted the native SP and framed *cfaC* to the lac SP. The PCR product was confirmed by DNA sequencing. The correct orientation of the *cfaC* following the NheI cloning was confirmed by restriction analysis and sequencing. This new component, Exp4-cfaA/lac-cfaC, was designed to have PmeI ends and therefore, it was moved into the PmeI site of pBzMM122. The correct orientation was validated by restriction enzyme analysis and confirmed by DNA sequencing. Finally, two extra Shine-Dalgarno sequences were inserted into a SpeI site and an ApaI site that were designed into pBzMM122 upstream of the canonical SD and immediately before the PmeI site, respectively. The whole cassette containing the four restructured genes was then moved as an AgeI fragment into the pMSP3535H3 backbone and placed under the control of Nisin-inducible and synthetic composite promoter (pBzMM156 and pBzMM153, respectively). It was also moved as a SmaI fragment under the control of the p23 promoter (pBzMM155) into a vector derived from pIB184 after adding a transcription termination signal.

### Immunoelectron Microscopy

Whole mount grids and ultrathin sections from TEM and high-pressure freeze and freeze substitution (HPF/FS) were immunolabeled at room temperature as follows: bacteria were applied to grids which were treated with 200mM NH_4_Cl in PBS for 20 min., rinsed with PBS, and incubated 1hr with blocking solution (1.5% BSA, 0.5% cold water fish skin gelatin, 0.01% Tween-20 in PBS [pH 7.2]). Grids were then incubated 1hr with in-house produced rabbit IgG anti-CFA/I fimbriae antibody diluted 1:100 in PBS, washed with PBS 3X, 5 minutes each, followed by 1hr incubation with 5nm colloidal gold conjugated goat-anti rabbit IgG (BBI Solutions, Madison WI USA) diluted 1:30 in PBS. Final washes were in PBS and distilled water and negatively stained with a 10 μl droplet of 2% aqueous uranyl acetate and Reynold’s lead citrate. Stain was removed by gently touching edge of filter paper and air dried. Ultrathin sections were examined using a Hitachi H-7000 TEM (Hitachi High Technologies America, Inc. Dallas, TX USA), and digital images acquired with a Veleta 2k x 2k camera and iTEM software (Olympus Soft-Imaging Solutions Corp. Golden, CO USA).

### Collagen-induced Arthritis (CIA) and Histopathology

C57BL/6 8- to 10-wk old males (Charles River Laboratories, Horsham, PA USA) were maintained at Montana State University Animal Resources Center or the University of Florida Animal Center Services. B6 mice were induced with CIA using 100 μg of chicken collagen II (CII; Chondrex, Redmond, WA USA) as previously described [19]. Each limb was evaluated using a scale of 0–3 [19]: 0, no clinical signs; 1, mild redness of a paw or swelling of single digits; 2, significant swelling of ankle or wrist with erythema; 3, severe swelling and erythema of multiple joints; maximum score per mouse is12. To treat CIA, groups of CIA mice were orally gavaged with sterile 50% saturated sodium bicarbonate solution followed by PBS, *L*. *lactis vector*, or *L*. *lactis*-CFA/I: a three-dose regimen involved treatments on days 14, 21, and 28; a two-dose regimen involved treatments on days 18 and 25 post-CIA induction. Clinical scores were measured in a double-blind fashion after treatment, and mice were monitored to day 40.

Paws from front and hind limbs and knees were prepared similar to that previously described using H&E to measure extent of inflammation or toluidine blue to determine the extent of cartilage matrix and chondrocyte loss [19–21].

### Anti-CFA/I and Anti-CII Endpoint Antibody Titers

Anti-CFA/I and anti-CII endpoint titers were done using ELISA methods [19–21].

### Cytokine ELISA

CD4^+^ T cells were cell-sorted by negative selection on magnetic beads (Invitrogen, Grand Island, NY USA) from lymph nodes (LNs) yielding purity >98%. Purified CD4^+^ T cells (10^6^/ml) were restimulated with 50 μg/ml CII (T-Cell Proliferation; Chondrex) for 4 days at 37°C and 5% CO_2_ in the presence of syngeneic irradiated (3000 rad) Ag-presenting cells. Cytokines in collected supernatants were measured by cytokine-specific ELISAs [19–21].

### Flow Cytometry and Adoptive Transfers

Splenic and LN cells were stained with fluorochrome-labeled mAbs to CD4, CD39, Ly-6G, Ly-6C, CD11b, and Foxp3 (eBioscience, San Diego, CA USA), TGF-β (R&D Systems, Minneapolis, MN USA), and fluorochrome-conjugated streptavidin (BD Pharmingen, San Jose, CA USA). Intracellular Foxp3 staining was done as previously described [19,21]. To measure inflammatory cells in the arthritic joints, isolated limb joints were digested with 2 mg/ml collagenase for 30 min at 37°C, and cell suspensions passed through 70 μm cell strainer. Cells were stained and analyzed by forward and side-scatter plots for Ly-6G^+^Ly-6C^+^CD11b^+^ neutrophils. For adoptive transfer of CD39^+^ CD4^+^ T cells, donor cells were obtained from B6 mice dosed thrice with 5×10^8^ CFUs *L*. *lactis*-CFA/I or *L*. *lactis* vector on days 0, 2, and 4, and on day 7, spleens and LNs were subjected to cell-sorting to procure >95% purity of CD39^-^CD4^+^ and CD39^+^CD4^+^ T cell subpopulations. Each T cell subset was adoptively transferred into separate groups of B6 recipients previously induced with CIA 14 days earlier. Recipients were monitored for disease.

### Statistics

Mann-Whitney *U* test was applied to statistically analyze clinical scores. Difference in arthritis incidence between experimental groups was checked with Fisher’s exact probability test. One-way ANOVA was performed to analyze ELISA and flow cytometry results. Data were considered statistically significant, if *p*-value was <0.05.

## Results and Discussion

Arthritis affects as many as 50 million adult patients in the US [3], and poses a substantial health-care burden, particularly because of its chronic nature, lessening the quality of life of these patients. RA is a chronic, systemic inflammatory disorder which is three times more common in women than men [4]. This disease primarily targets the synovial joints, where tissue destruction is mediated in part by the overexpression of inflammatory cytokines [5], and approximately 42% of the affected patients have doctor-diagnosed arthritis and arthritis-attributable activity limitation (AAAL) [3]. Dependence on chronic use of anti-inflammatory interventions for RA patients is problematic, particularly with treatments involving targeted suppression of TNF-α which often results in increased susceptibility to infections [2, 9, 10]. Hence, intervention strategies that redirect T cell responses may be more effective especially for the long-term.

CFA/I fimbriae are encoded by an operon containing 4 structural genes whose products are all secreted and eventually assemble into polymerized fimbriae at the outer membrane [15,22]. CFA/I fimbriae are heteropolymers composed of a tip protein, the minor adhesive subunit, CfaE, joined to a shaft of about ~1000 major subunits, CfaB. Assembly is coordinated by CfaA, a periplasmic chaperone which directs the proper folding, polymerization, and delivery to the outer membrane usher protein. CfaC ushers the subunits and acts as a platform to enable proper assembly of the fimbriae into their helical structure [23]. Originally designed as a diarrheal vaccine for enterotoxigenic *E*. *coli* when expressed by *Salmonella* [16], unexpectedly, the CFA/I fimbriae also induced fimbriae-specific Tregs [19, 24]; however, these Tregs did not blunt the vaccine’s capacity to protect against infectious diseases [25]. Given these properties, we hypothesized that CFA/I fimbriae in a bystander fashion, can suppress inflammatory diseases. In testing this hypothesis, it became evident that disease-specific Treg cells were also induced in experimental animal models for RA [19] and multiple sclerosis [24]. However, given the limitations of using *Salmonella*-based therapeutics in humans, a benign method for delivering the CFA/I fimbriae was sought. The food-grade Gram-positive *Lactococcus lactis* was selected since it is commonly used in fermented dairy products [13], approved for use in humans, successfully adapted for delivering IL-10 in colitis patients [26], and it does not colonize the intestine [27] allowing for time-limited and precise dosing.

**Fig 1:**
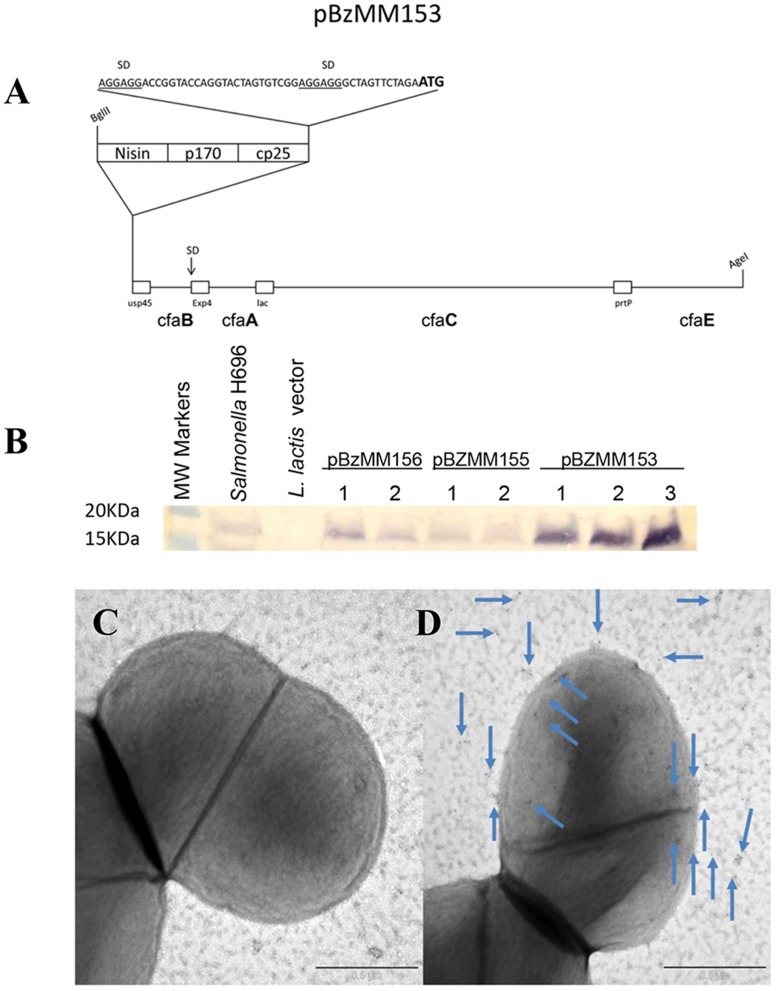
Expression of *E*. *coli cfaI* fimbrial operon in *Lactococcus lactis*. **(A)** Schematic map for expression of *E*. *coli cfaI* in *L*. *lactis* (pBzMM153). The elements of the composite promoter are boxed. Each structural gene is fused in-frame to a different lactococcal secretion signal peptide, and each gene is followed by its own STOP codon. No other functional elements are interspersed except for one Shine-Dalgarno (SD) sequence upstream of a fusion between extracellular (Exp4) protein and *cfaA*. All (SD) sequences are marked. **(B)** CFA/I fimbriae expression in *L*. *lactis*. Lane1, molecular weight (MW) markers; lane 2, *Salmonella*-CFA/I (H696) strain; lane 3, *L*. *lactis* bearing the empty pMSP3535H3 vector; lanes 4,5, pBzMM156 (nisin-inducible promoter) clones 1 and 2; lanes 6,7, pBzMM155 (p23 promoter) clones 1 and 2; and lanes 8–10, pBzMM153 (synthetic composite promoter) clones 1–3. **(C, D)** Immunogold staining of **(C)** *L*. *lactis* vector and **(D)** *L*. *lactis*-CFA/I with anti-CFA/I antibody. Arrows point to gold particles on fimbrial structures projecting from the cell wall.

To adapt the CFA/I fimbrial operon, it was reengineered for expression in *L*. *lactis* subsp *lactis* IL1403. Expression of CFA/I fimbriae in *Lactococcus lactis* ssp. *lactis* IL1403 was achieved via sequential optimizations using a combination of genetic engineering techniques and synthetic biology. The operon was placed under the control of different promoters, including a semi-synthetic one that we designed *in silico* to achieve the best expression. This promoter drives a substantially higher expression when compared to both nisin-inducible and p23 promoters, which are amongst the strongest lactococcal promoters available. The construct with the semi-synthetic promoter, termed pBzMM153, showed the greatest CfaB expression, and it was used to generate *Lactococcus*-pBzMM153 (Fig. 1A, B). Retention of pBzMM153 in milk without selection was >88% over 16 generations, compared to 100% for the *Lactococcus* vector strain bearing pMSP3535H3 (data not shown). Attempts to remove *cfaC*, which facilitates the export of fimbrial subunit outside the *E*. *coli* outer cell membrane and orchestrates the polymerization of the fimbriae, failed to express any detectable CfaB major subunit (data not shown). Upon examination by immuno-electron microscopy, immunogold particles label fimbrial structures projecting from the surface of *Lactococcus*-CFA/I (Fig. 1D), whereas the *Lactococcus* vector control strain does not stain (Fig. 1C).

**Fig 2:**
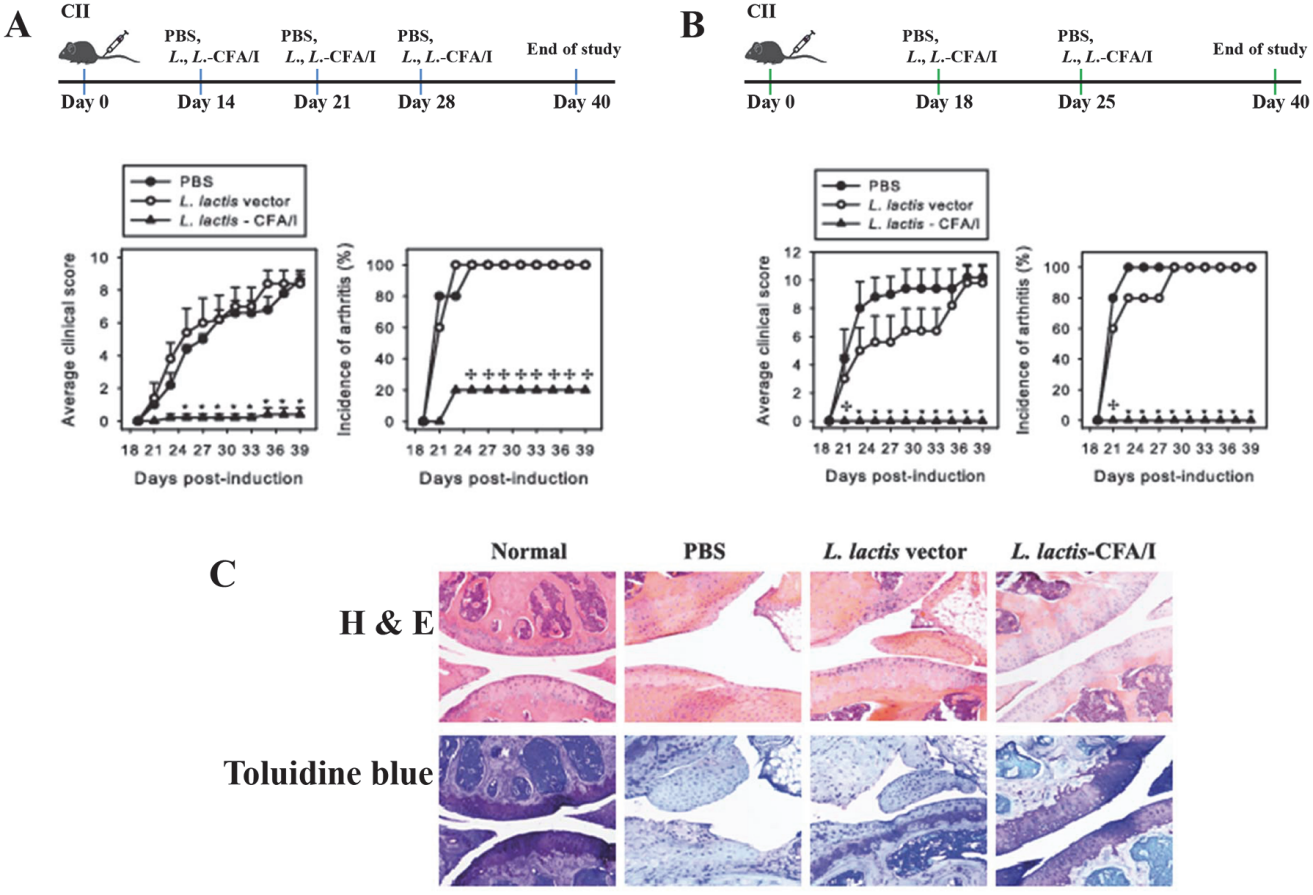
*L*. *lactis*-CFA/I, not *L*. *lactis* vector, is a potent therapeutic for collagen-induced arthritis (CIA). C57BL/6 males (n = 5/group) were CII-challenged on day 0, and treated orally with 5×10^8^ CFUs *L*. *lactis*-CFA/I (*L*. *lactis*-pBzMM153) or vector control (*L*. *lactis*-pMSP3535H3) in sterile PBS or with vehicle alone on days **(A)** 14, 21, 28 or **(B)** on days 18 and 25 post-CIA induction as diagramed. Bacteria were grown in synthetic M17 medium supplemented with 0.5% glucose. A representative example of 6 experiments (n = 5/group) is depicted; * *p* < 0.01; ^✢^ *p* < 0.05 as compared to each control group. **(C)** Joint pathology was evaluated from decalcified knees from mice in each treatment group (n = 5/group). Representative examples of mid-sagittal knee joint sections are stained with H&E (top row) or toluidine blue (bottom row) from mice treated with two doses of *L*. *lactis*-CFA/I **(B)** upon termination of the study.

**Fig 3:**
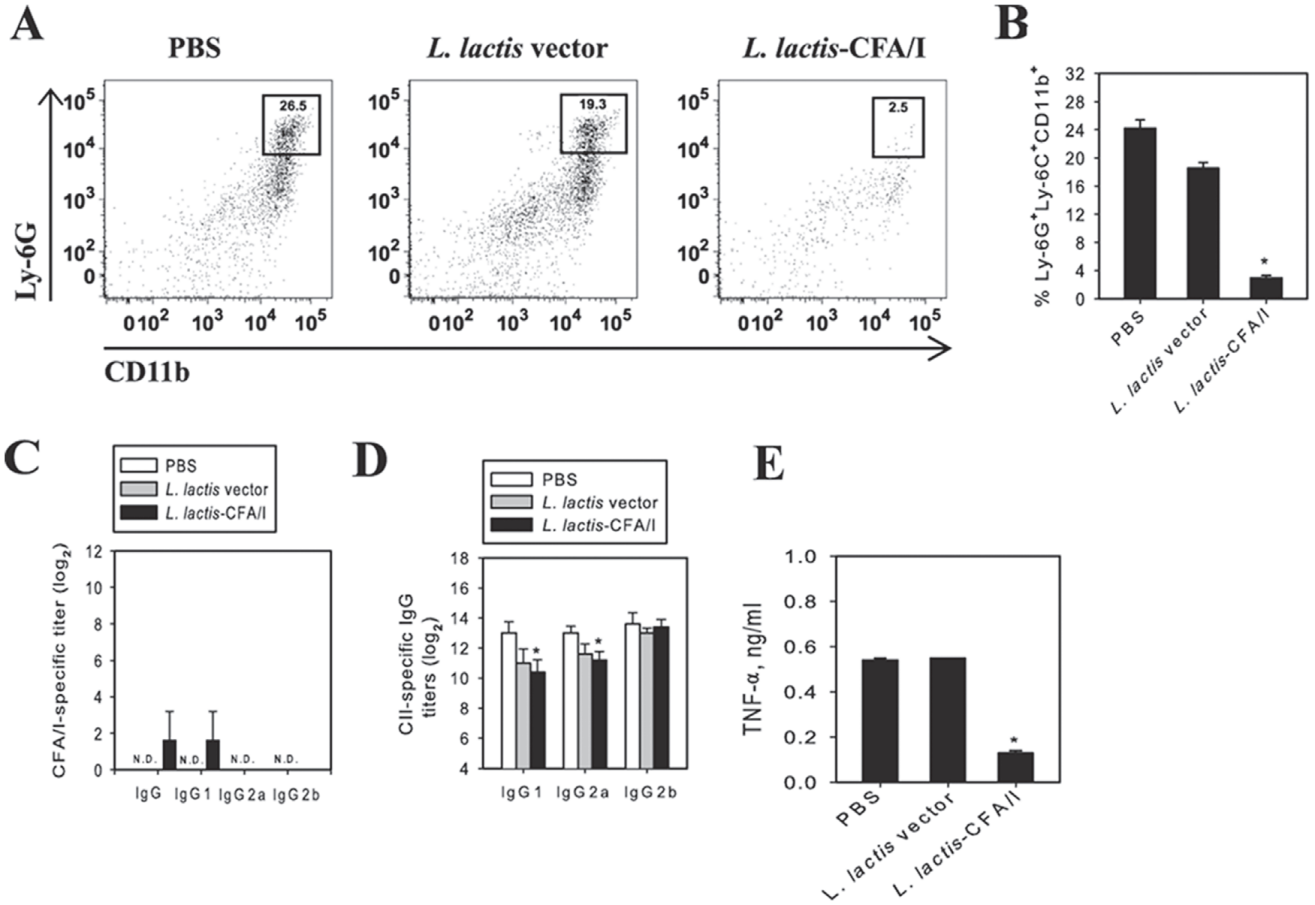
*L*. *lactis*-CFA/I confers protection against CIA via reduction of neutrophil influx into joints with concomitant reduction in TNF-α, but only slightly reduces anti-CII Ab titers. Reduced tissue destruction in Fig. 2 is attributed to diminished neutrophil infiltration of the joints **(A, B)** in *L*. *lactis*-CFA/I-treated mice (as described in Fig. 2B). Cell suspensions were analyzed by flow cytometry for Ly-6G^+^Ly-6C^+^CD11b^+^ neutrophils; * *p* < 0.001 compared with control groups (n = 5/group). **(C)** *L*. *lactis*-CFA/I fails to elicit serum IgG and IgG subclass anti-CFA/I fimbriae and **(D)** modestly reduces IgG1 and IgG2a anti-CII antibody titers (day 35 post-CIA induction) in orally treated mice from Fig. 2; n = 5 mice/group. **(E)** LN lymphocytes from *L*. *lactis*-CFA/I-treated mice from Fig. 2B (n = 5 mice/group) were CII-restimulated in vitro and showed reduced TNF-α production; * *p* < 0.001 compared to each control group.

To test its therapeutic properties, mice were subjected to CIA, a rodent model that shares attributes associated with RA [6,8]. Given this similarity, CIA is often exploited as a tool to investigate novel strategies and therapeutics to prevent and treat RA. Mice were challenged with CII on day 0 [19], and then were randomly divided into three groups for oral treatment with *L*. *lactis*-CFA/I (pBzMM153), *L*. *lactis* vector (pMSP3535H3), or with sterile PBS. Two treatment paradigms were tested: beginning intervention on day 14 resembling *Salmonella*-CFA/I treatment [19] with two additional doses on days 21 and 28 (Fig. 2A) or beginning intervention at disease onset on days 18 and 25 (Fig. 2B). Using the three-dose regimen, only 20% of the *L*. *lactis*-CFA/I-treated mice showed mild redness of their paws as opposed to PBS- or *L*. *lactis* vector-treated mice, who all developed arthritis by day 19 post-CII challenge eventually achieving clinical scores of ~9 (Fig. 2A). To test if only two doses of *L*. *lactis*-CFA/I would also protect against CIA, groups of CII-challenged mice were treated at the onset of disease with a second dose given 1 wk later. None of the *L*. *lactis*-CFA/I-treated mice developed disease unlike those treated with PBS or *L*. *lactis* vector (Fig. 2B). Histopathological analysis of H&E-stained joint sections showed significantly less inflammation and joint destruction confirming these clinical findings of the two-dose treatment regimen (Fig. 2C). Moreover, *L*. *lactis*-CFA/I-treated mice exhibited no chondrocyte or proteoglycan matrix loss as evident from the toluidine blue-stained sections unlike the severe cartilage degeneration and bone erosion observed in PBS- and *L*. *lactis* vector-treated mice (Fig. 2C). Inflammation was also assessed by measuring the extent of neutrophil infiltration of the joints (Fig. 3A, B). In agreement with clinical and histopathological findings, *L*. *lactis*-CFA/I-treated mice revealed virtually no Ly-6G^+^ CD11b^+^ cells which amounted to greater than an 8-fold reduction relative to PBS-treated mice. Consistent with *Lactococcus* transient presence [27], no anti-CFA/I fimbriae antibodies were detected (Fig. 3C), and anti-CII antibodies were modestly reduced (Fig. 3D). CII-restimulated lymphocytes obtained from the draining LNs showed a remarkable suppression (>4.2-fold) of disease-promoting TNF-α in *L*. *lactis*-CFA/I-treated mice relative to PBS- or *L*. *lactis* vector-treated mice (Fig. 3E). Thus, these studies demonstrate that the engineered *L*. *lactis*-CFA/I is an effective therapeutic capable of inhibiting arthritis by suppressing TNF-α production and inhibiting neutrophil influx into the inflamed joints abating tissue destruction.

**Fig 4:**
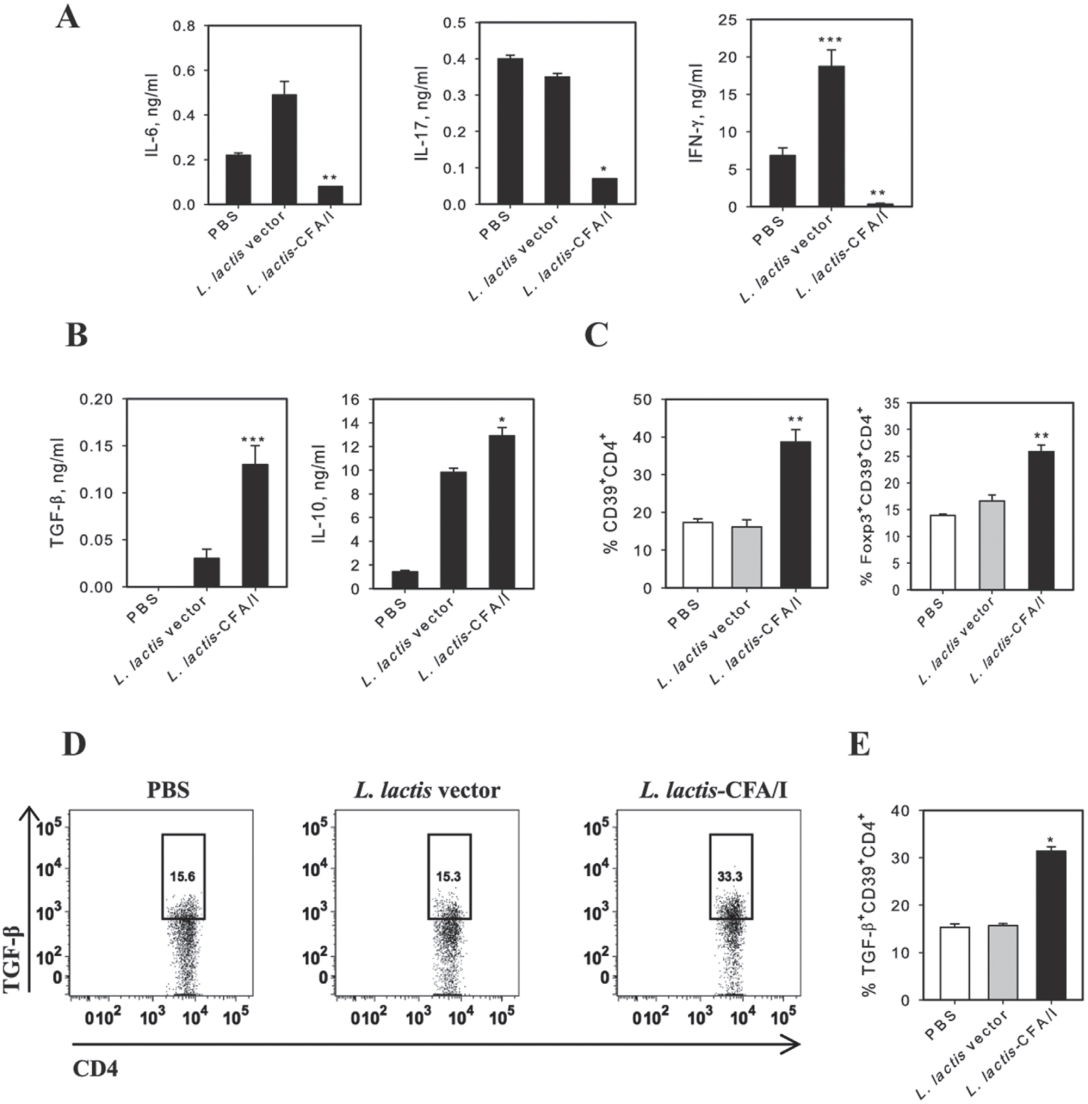
*L*. *lactis*-CFA/I reduces CII-specific inflammatory CD4^+^ T cells with concomitant increases in anti-inflammatory cytokines. On day 40 post-CIA induction, purified LN CD4^+^ T cells from each group were restimulated with 50 μg/ml CII for 4 days in presence of syngenic irradiated Ag-presenting cells. **(A)** *L*. *lactis*-CFA/I, not *L*. *lactis* vector, suppress IL-6, IL-17, and IFN-γ responses and show elevated **(B)** TGF-β and IL-10. Depicted are the means ± SD of triplicate cultures as assessed by cytokine-specific ELISA; one of 3 experiments (5 mice/group) is shown. **(C)** Frequency of CD39^+^CD4^+^ T cells in LNs of control and protected mice (5 mice/group). **(D, E)** Expression of TGF-β for gated LN CD39^+^CD4^+^ T cells mice treated with PBS, *L*. *lactis* vector, or *L*. *lactis*-CFA/I. Representative FACS plots **(D)** and frequencies of TGF-β^+^CD39^+^CD4^+^ T cells **(E)** are depicted. For all studies, *p < 0.001, **p < 0.005, and ***p < 0.05 as compared with PBS- and *L*. *lactis* vector-treated mice.

**Fig 5:**
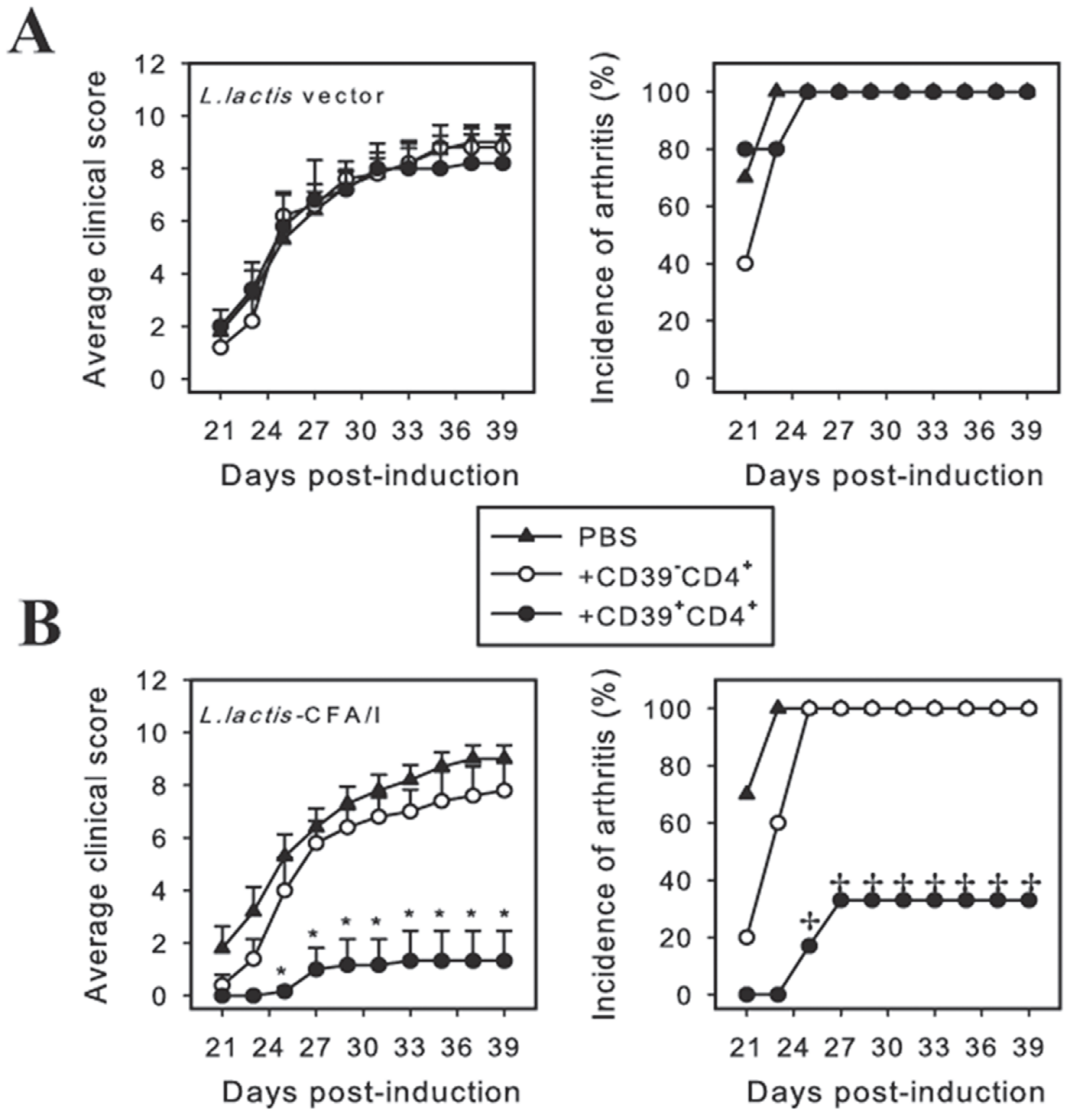
*L*. *lactis*-CFA/I-induced CD39^+^CD4^+^ T cells protect against CIA. Donor mice were treated with *L*. *lactis*-CFA/I or *L*. *lactis* vector on days 0, 2, and 4. Three days after the last dose, spleens and LNs were harvested for cell-sorting of CD39^-^CD4^+^ and CD39^+^CD4^+^ T cells, and adoptively transferred into recipients previously CII-challenged 14 days earlier (n = 6–10 mice/group). Severity and incidence of disease in CIA recipients of **(A)** *L*. *lactis* vector- and **(B)** *L*. *lactis*-CFA/I-induced CD39^-^CD4^+^ and CD39^+^CD4^+^ T cells are shown; *p < 0.005; ^✢^ *p* < 0.01 as compared with PBS- treated mice and with CD39^-^CD4^+^ T cell recipients.

Given the robust protection conferred by *L*. *lactis*-CFA/I, we queried whether the CFA/I fimbriae impacted CD4^+^ T cells since these are culpable for eventual tissue destruction in arthritis. At the termination of the study, CD4^+^ T cells were isolated from draining axillary, popliteal, and inguinal LNs from each treatment group, restimulated with CII, and the cytokine expression pattern was measured. Consistent with clinical scores, LNs from *L*. *lactis*-CFA/I-treated mice showed a marked reduction in IL-6, IL-17, and IFN-γ production (Fig. 4A) with concomitant increases in the anti-inflammatory cytokines, TGF-β and IL-10 (Fig. 4B). Similar cytokine profiles were obtained from CIA mice treated once with the *L*. *lactis* therapeutics and measured one wk after treatment (data not shown). These anti-inflammatory cytokines are generally produced by Foxp3^+^ CD4^+^ T cells (Tregs), which have a pivotal role in dampening autoimmune diseases [28]. Unlike other autoimmune diseases whose Tregs are CD25^+^ [24], we have found that the primary Tregs responsible for resolving arthritis are CD39^+^ [19]. CD39, an ectonucleoside triphosphate diphosphohydrolase-1, has the advantage of hydrolyzing ATP into AMP enabling the *L*. *lactis*-CFA/I-induced Tregs to quench inflammatory signaling by extracellular ATP [29, 30]. To assess if *L*. *lactis*-CFA/I stimulated such Tregs, flow cytometry analysis of stained LN lymphocytes was performed, and revealed that CD39^+^ Tregs were induced and the amount of CD39^+^ Tregs relative to those induced by *L*. *lactis* vector- or PBS-treated mice doubled (Fig. 4C). Of these Tregs, nearly 30% was Foxp3^+^ (Fig. 4C), and 75% of these were IL-10^+^ (data not shown). *L*. *lactis*-CFA/I also induced a subset of TGF- β-producing CD39^+^CD4^+^ T cells, mostly Foxp3^-^ (Fig. 4D, E). To assess the functionality of the observed CD39^+^ Tregs upon autoimmune disease, an adoptive transfer study was performed. CD39^+^ CD4^+^ and CD39^-^ CD4^+^ T cells were cell-sorted from donors previously dosed three times with *L*. *lactis*-CFA/I or *L*. *lactis* vector, and adoptively transferred into recipients previously challenged with CII 14 days earlier. Donor CD39^+^ CD4^+^ and CD39^-^ CD4^+^ T cells obtained from *L*. *lactis* vector-treated mice failed to protect CIA mice, and incidence of disease was 100% (Fig. 5A). In contrast, CIA recipients given donor CD39^+^ CD4^+^ T cells, not CD39^-^ CD4^+^ T cells, obtained from *L*. *lactis*-CFA/I-treated mice demonstrated attenuation of CIA with reduction in both disease incidence and disease severity (Fig. 5B). Thus, these studies show that *L*. *lactis*-CFA/I-induced CD39^+^ CD4^+^ T cells are protective against CIA.

**Fig 6:**
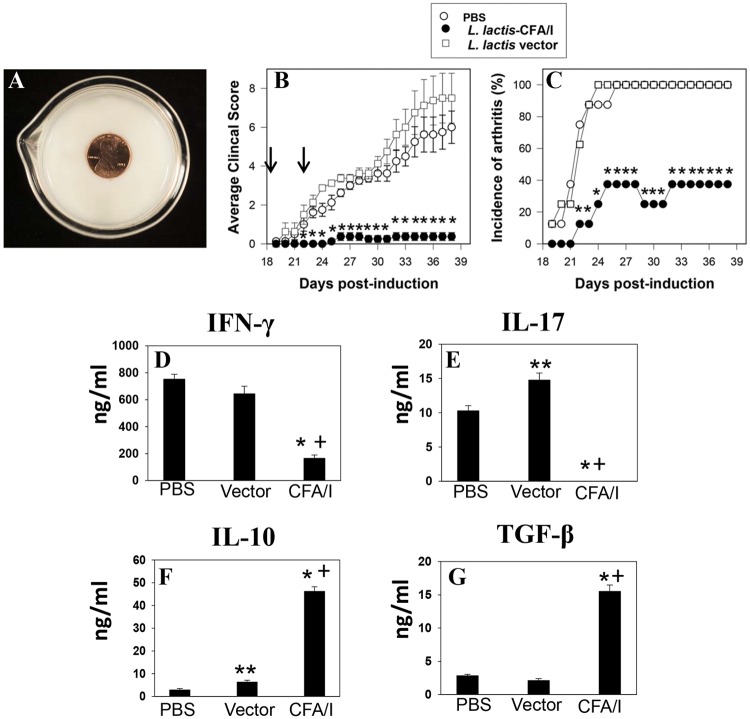
Two doses of *L*. *lactis*-CFA/I fermented milk protect against CIA. Groups of C57BL/6 mice (n = 8/group) were CII-challenged on day 0, and treated twice on days 18 and 22 with **(A)** *L*. *lactis*-CFA/I- or *L*. *lactis* vector-fermented milk that contained 2.5×10^8^ CFUs or treated with sterile PBS. Mice were monitored for disease until day 39 measuring **(B)** average clinical score **(C)** and incidence of arthritis. Arrows indicate days of fermented milk administration; * *p* ≤ 0.001 as compared to each control group. **(D-G)** LN CD4^+^ T cells isolated from each treatment group were restimulated with 50 μg CII in the presence of mitomycin C-treated Ag-presenting cells for 4 days. CIA mice treated with *L*. *lactis*-CFA/I fermented milk showed reduced LN **(D)** IFN-γ and **(E)** IL-17 production with concomitant increases in **(F)** IL-10 and **(G)** TGF-β; * *p* ≤ 0.001, ** *p* < 0.05 versus PBS-treated mice; and ^*+*^ *p* ≤ 0.001 versus *L*. *lactis* vector-treated mice.

An advantage of modifying *L*. *lactis* species is its future application in treating arthritis and other autoimmune diseases when given to patients in a fermented dairy product. Since *L*. *lactis* is commonly used in fermented dairy products [13], we queried whether *L*. *lactis* fermented milk could be used to treat CIA mice. *L*. *lactis* strains were grown in pasteurized, fortified whole milk with bacterial growth densities similar to *L*. *lactis* strains grown in synthetic M17 glucose medium, resulting in a semisolid, yogurt-like texture with a pH of 4.8 (Fig. 6A) similar to commercially prepared yogurts. Moreover, the lactococci were much more stable in the fermented milk unwavering in their viability for at least two weeks unlike those lactococci grown in M17 medium whose viability declined after 2 days (data not shown). To test the therapeutic’s efficacy, groups of mice previously CII-challenged were orally gavaged with *L*. *lactis*-CFA/I- or *L*. *lactis* vector-fermented milk at the onset of clinical signs of CIA and 4 days later. One group of mice received PBS only. Both groups receiving either PBS or *L*. *lactis* vector-fermented milk all eventually developed arthritis by day 26 post-CII challenge, and clinical scores between these two groups were not significantly different (Fig. 6B, C). Thus, treatment of CIA mice with *L*. *lactis* fermented milk alone was ineffective in delaying disease onset, reducing disease incidence, or dampening clinical severity. In contrast, treatment of CIA mice with *L*. *lactis*-CFA/I fermented milk greatly ameliorated clinical severity of disease. Only 37.5% of the mice showed signs of arthritis, but these had remarkably reduced clinical score of 1 (Fig. 6B, C). Evaluation of their CD4^+^ T cell responses revealed that mice treated with *L*. *lactis*-CFA/I fermented milk showed greatly reduced IFN-γ and IL-17 (Fig. 6D, E) with elevated IL-10 and TGF-β (Fig. 6F, G) production confirming *L*. *lactis*-CFA/I’s ability to suppress inflammatory cytokines with the simultaneous capacity to augment IL-10 and TGF-β even when *L*. *lactis*-CFA/I is present in fermented milk.

In summary, what makes this work unique is the following: 1) the entire operon from a Gram-negative microorganism can be conformed into a Gram-positive host enabling the four genes to interact and produce functional, secreted fimbriae; 2) the data prove that a lab-adapted microorganism derived from an industrially relevant strain maintained its capacity to ferment milk into a stable yogurt-like dairy product, while retaining its desired therapeutic properties; and 3) the data promote the novel concept that bacterial fimbriae, though generally considered as virulence factors, can be exploited as therapeutic instruments to modify the host’s immune system to counteract inflammation. With respect to the latter, the potency of CFA/I fimbriae when delivered by *Lactococcus* to suppress arthritis was enhanced especially when compared to the potency of *Salmonella*-CFA/I intervention. A more dramatic reduction in CIA was observed with the *Lactococcus* delivery platform than *Salmonella* [19,31]. These data show that oral *L*. *lactis*-CFA/I greatly ameliorates CIA both when grown in synthetic medium or in milk. *L*. *lactis*-CFA/I mediates its protection via the stimulation of CD39^+^ Tregs producing IL-10 and TGF-β with concomitant suppression of inflammatory cytokines, particularly TNF-α, and inhibition of neutrophil influx into affected joints. Such protective properties against arthritis were not evident with PBS- or *L*. *lactis* vector-treated mice. Importantly, this protection is rendered in the absence of disease Ag specificity, greatly facilitating this therapeutic for treating multiple autoimmune and inflammatory diseases.
